# Wnt3a protects SH-SY5Y cells against 6-hydroxydopamine toxicity by restoration of mitochondria function

**DOI:** 10.1186/s40035-015-0033-1

**Published:** 2015-06-16

**Authors:** Lei Wei, Li Ding, Ming-shu Mo, Ming Lei, Limin Zhang, Kang Chen, Pingyi Xu

**Affiliations:** Department of Neurology, The Third Affiliated Hospital of Sun Yat-sen University, Guangzhou, 510630 China; Department of Neurology, The First Affiliated Hospital of Sun Yat-sen University, Guangzhou, 510080 China; Department of pathology, The First Affiliated Hospital of Sun Yat-sen University, Guangzhou, 510080 China; Department of Neurology, The First Affiliated Hospital of Guangzhou Medical University, Guangzhou, 510120 China; Division of Clinical Laboratory, Zhongshan Hospital of Sun Yat-sen University, Zhongshan, 528403 China

**Keywords:** Wnt3a, 6-OHDA, Mitochondria function, Parkinson’s disease

## Abstract

**Background:**

Wnt/β-catenin signal has been reported to exert cytoprotective effects in cellular models of several diseases, including Parkinson’s disease (PD). This study aimed to investigate the neuroprotective effects of actived Wnt/β-catenin signal by Wnt3a on SH-SY5Y cells treated with 6-hydroxydopamine (6-OHDA).

**Methods:**

Wnt3a-conditioned medium (Wnt3a-CM) was used to intervene dopaminegic SH-SY5Y cells treated with 6-OHDA. Cell toxicity was determined by cell viability and lactate dehydrogenase leakage (LDH) assay. The mitochondria function was measured by the mitochondrial membrane potential, while oxidative stress was monitored with intracellular reactive oxygen species (ROS). Western blot analysis was used to detect the expression of GSK3β, β-catenin as well as Akt.

**Results:**

Our results showed that 100 μM 6-OHDA treated for 24 h significantly decreased cell viability and mitochondrial transmembrane potential, reduced the level of β-catenin and p-Akt, increased LDH leakage, ROS production and the ratio of p-GSK3β (Tyr216) to p-GSK3β (Ser9). However, Wnt3a-conditioned medium reversing SH-SY5Y cells against 6-OHDA-induced neurotoxicity by reversing these changes.

**Conclusions:**

Activating of Wnt/β-catenin pathway by Wnt3a-CM attenuated 6-OHDA-induced neurotoxicity significantly, which related to the inhibition of oxidative stress and maintenance of normal mitochondrial function.

## Background

Parkinson’s disease (PD), characterized by loss of dopaminergic neurons in substantia nigra, is the second most common neurodegenerative diseases in elderly people. Studies have shown that several mechanisms are involved in pathogenesis of PD including oxidative stress, mitochondrial dysfunction and elevated brain iron levels [1–3]. Although much research has helped to elucidate the pathogenesis of PD, the precise etiology and pathogenesis of the disease still remain unknown. Moreover, Current treatments for PD predominantly rely on pharmacotherapy to improve the symptoms of movement disorder, but little efficacy in preventing disease progression. Therefore neuroprotective therapy may play a key role in the therapeutic strategy of PD.

Wnt signaling pathway is an autocrine-paracrine signal transduction pathway which has been demonstrated to participate in embryonic development, cell differentiation and ontogenesis [4–7]. A main Wnt signaling pathway branch is the wnt/β-catenin pathway, which initiates with Wnt proteins binding to Frizzled receptors and activates Dishevelled. Activation of Dishevelled results in inhibition of glycogen synthase kinase-3β (GSK3β), which in turn causes stabilization of β-catenin. Stabilized β-catenin accumulates and is taken into the nucleus where it regulates expression of numerous genes [8]. Extensive research has confirmed the vital role of Wnt signaling in midbrain dopaminergic neuronal development [9, 10]. For example, Wnt1 and Wnt3a, which exert effects by Wnt/β-catenin pathway, are key regulators in the development of dopaminergic neurons [9].

The cellular protective effects of Wnt/β-catenin pathway have been demonstrated in animal and cellular models of Alzheimer’s disease, retinal degeneration, cerebral ischemia as well as PD [11–14]. Our previous study hasdemonstrated that Wnt/β-catenin signal is inhibited in SH-SY5Y cells treated with 6-OHDA, a cellular model of PD, while activation of Wnt/β-catenin signal by exogenous Wnt1 could protect cells by restoring mitochondria and endoplasmic reticulum function [15]. However, the precise mechanism for the pathogenesis of the disease remains unknown. In this study, we report that Wnt3a-conditioned medium (Wnt3a-CM) protected cells from 6-OHDA neurotoxicity by a mechanism that involved maintenance of normal mitochondrial.

## Methods

### Cell culture

Human neuroblastoma SH-SY5Y cells were obtained from American Type Culture Collection (ATCC, Manassas, VA, U.S.A.), maintained in DMEM with high glucose (Invitrogen, USA) supplemented with 10 % fetal bovine serum (FBS, Invitrogen), and cultured in a humidified incubator with 5 % CO_2_ at 37 °C. Cells with 20–30 passages were used. For experiments, cells were seeded at a density of 1 × 10^5^ /cm^2^ in the plastic flasks or plates. Conditioned media containing Wnt3a (Wnt3a-CM) were prepared from mouse L cells (ATCC) stably expressing Wnt3a. Control conditioned media were obtained from parental L cells (Ctrl-CM). Different proportions of Ctrl-CM or Wnt3a-CM were performed according to corresponding experiments.

### Cell viability assay

SH-SY5Y cells were seeded in a 96-well plate at a density of 1 × 10^3^ cells per well. After attachment, cells were treated with 100 μM 6-OHDA, Ctrl-CM (10–80 %) or Wnt3a-CM (10–80 %) for 24 h. After treatment, cells were incubated with 0.5 mg/mL MTT (Sigma-Aldrich, USA) for 4 h at 37 °C [16]. Following aspiration of the MTT solution, the same volume of DMSO was added into each well to dissolve the purple formazan crystals. Absorbance was read in a microtiter plate reader at 490 nm. Cell viability was expressed as a percentage of the absorbance from control cells. The toxic effects of 6-OHDA to SH-SY5Y cells were also detected by measuring the leakage of the cytosolic enzyme LDH to the culture medium using a colorimetric LDH assay kit (KeyGen, China) according to the manufacturer’s instructions [17]. Briefly, after 100 μM 6-OHDA added, 20 μl of cell medium were added into basic solution to measure extracellular LDH activity, which could catalyze the conversion of lactate to pyruvate, which then reacted with 2,4-dinitrophenylhydrazine to give the brownish red color. The absorbance was measured at a wavelength of 440 nm by colorimetric assay, and the LDH leakage was expressed as the percentage versus control cells.

### Measurement of mitochondrial transmembrane potential (MMP) and intracellular ROS production

Changes in the mitochondrial membrane potential with various treatments in SH-SY5Y cells were measured by rhodamine-123 or DCFH-DA using a fluorescence spectrophotometer [18]. Briefly, cells were treated with 6-OHDA, Ctrl-CM and Wnt3a-CM for 24 h and then incubated with rhodamine-123 (Sigma-Aldrich, USA) or DCFH-DA (Sigma-Aldrich, USA) in a final concentration of 10 or 25 μmol/L respectively for 30 min at 37 °C. After washing twice with HEPES buffer saline (Invitrogen, USA), fluorescence was recorded at 488 nm excitation and 523 nm emission wave-lengths. Each field of cells was photographed using a fluorescence microscopy.

### Western blot analysis

The immunoblotting was performed in accordance with a standard procedure [16, 19]. The following primary antibodies were used: rabbit anti-β-catenin (1:1000 dilution, Abcam, Cambridge, UK), rabbit anti-p-GSK3β (Ser9) (1:1000 dilution, CST, USA), rabbit anti-p-GSK3β (Tyr216) (1:1000 dilution, CST, USA), rabbit anti-p-Akt (Ser473) (1:1000 dilution, Millipore, USA), mouse anti-β-actin (1:1000 dilution, Millipore, USA). Proteins were detected with the SuperSignal® West Pico Chemiluminescent Substrate (Thermo Fisher Scientific Inc., IL, USA) and membranes were exposed to X-ray films (Fujifilm Corporation, Japan), which were scanned and analyzed by using the Quantity One v4.62 for Windows software (Biorad, CA, USA).

### Statistical analysis

Results were presented as mean ± SD. One-way analysis of variance (ANOVA) followed by Student–Newman–Keuls test was used to compare differences between means in more than two groups. The level of significance was set at *P* < 0.05. All the statistical analyses were performed with SPSS 12.0 for windows (SPSS Inc., Chicago, IL, USA).

## Results

### Wnt3a-CM attenuated 6-OHDA-induced cell injury

Our previous studies have confirmed that treatment with 6-OHDA for 24 h caused a concentration-dependent reduction in cell viability and a concentration-dependent increase in LDH release in dopaminergic SH-SY5Y cells [15]. According to our previous results, a 100 μM 6-OHDA was chosen for the following experiments.

**Fig. 1:**
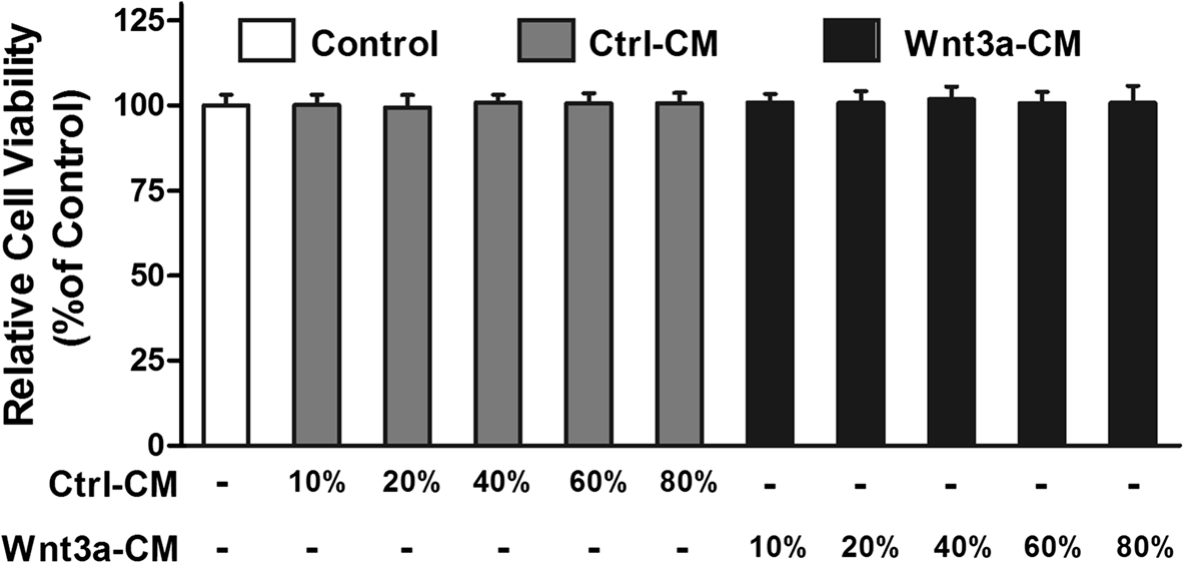
Treatment with Wnt3a-CM or Ctrl-CM didn’t change SH-SY5Y cell viability. SH-SY5Y cells were treated with different proportion of Ctrl-CM or Wnt3a-CM, cell viability was measured by MTT assay. Data were presented as mean ± SD from four independent experiments

**Fig. 2:**
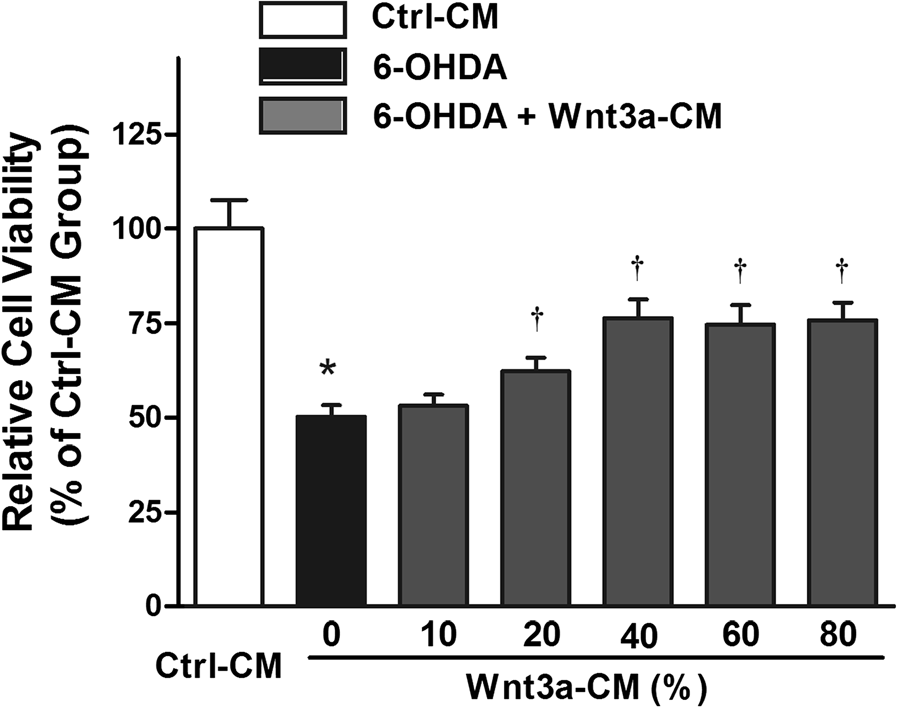
Effects of Wnt3a-CM on viability of SH-SY5Y cells treated with 6-OHDA. SH-SY5Y cells were pretreated with different proportion of Wnt3a-CM (10–80 %) prior to 6-OHDA (100 μM) treatment for 24 h and cell viability assessed using the MTT assay. The data are expressed as percentage relative to Ctrl-CM group and presented as mean ± SD from four independent experiments. *P < 0.05 compared to the control, †*P* < 0.05 compared to 6-OHDA treated group

**Fig. 3:**
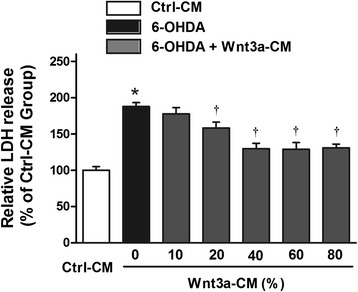
Effects of Wnt3a-CM on LDH leakage of SH-SY5Y cells treated with 6-OHDA. SH-SY5Y cells were pretreated with different proportion of Wnt3a-CM (10–80 %) prior to 6-OHDA (100 μM) treatment for 24 h and LDH assay was performed to determine the degree of cell injury. The data are expressed as percentage relative to Ctrl-CM group and presented as mean ± SD from four independent experiments. *P < 0.05 compared to the control, †*P* < 0.05 compared to 6-OHDA treated group

We then tested the effect of Wnt3a-CM on SH-SY5Y cells, and found that treatment with Wnt3a-CM or Ctrl-CM at proportion of 10–80 % didn’t obviously change the cell viability (Fig. 1). Thus, Ctrl-CM at a proportion of 40 % was used as control and we investigated whether Wnt3a-CM could attenuate the toxic effect of 6-OHDA on SH-SY5Y cells. Wnt3a-CM was added to the cultures at different proportion 20 min prior to 6-OHDA. Cells pre-treated with Wnt3a-CM were partially protected against 6-OHDA toxicity. Treatment with 100 μM 6-OHDA for 24 h decreased the cell viability to ~50 % compared with Ctrl-CM group. However, when cells were pre-treated with Wnt3a-CM the reduction of cell viability was ameliorated. Specifically, the level of cell viability increased to 62.16 ± 3.71 % of the control value when 20 % of Wnt3a-CM was used and that was up to 76.35 ± 5.00 % when 40 % of Wnt3a-CM was added. Then the cell viability of higher proportion of Wnt3a-CM (60 and 80 %) was reduced to 74.64 ± 5.21 and 75.79 ± 4.71 respectively (Fig. 2). Similarly, pretreatment of 20, 40, 60 and 80 % Wnt3a-CM could significantly inhibit LDH release induced by 6-OHDA (Fig. 3).

### Wnt3a-CM antagonized 6-OHDA-induced MMP and ROS production

**Fig. 4:**
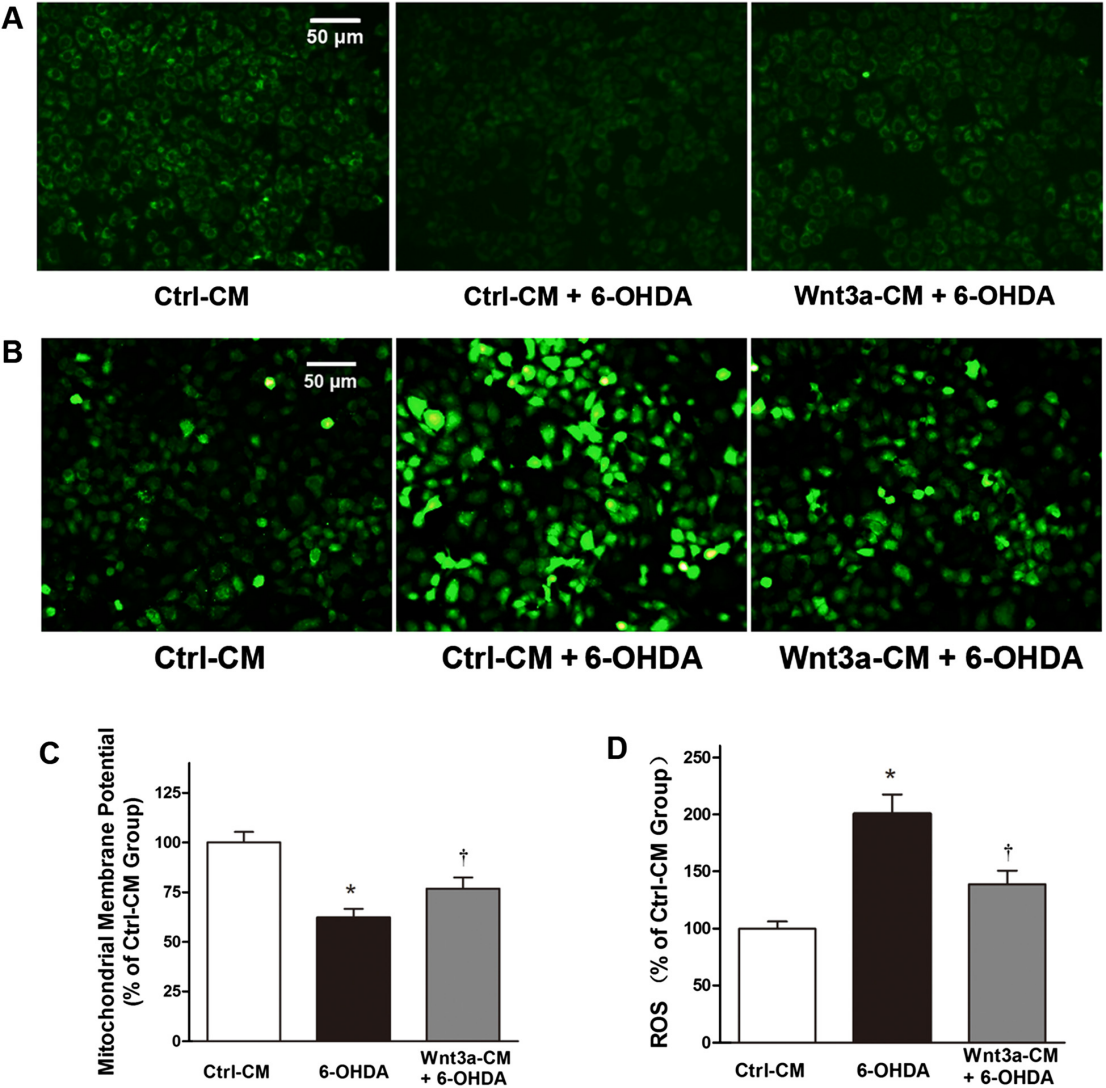
Mitochondrial membrane potential (MMP) AND intracellular ROS production. SH-SY5Y cells were treated with Ctrl-CM, Ctrl-CM + 6-OHDA (100 μM) or Wnt3a-CM (40 %) 20 min prior to 6-OHDA for 24 h, MMP (**a**) and intracellular ROS (**b**) were photographed by a fluorescence microscopy. Results of MMP (**c**) and intracellular ROS (**d**) are detected by a fluorescence spectrophotometer and expressed as relative fluorescent intensity. Data were presented as mean ± SD from four independent experiments. *P < 0.05 compared to Ctrl-CM group, †*P* < 0.05 compared to Ctrl-CM + 6-OHDA group

It is generally accepted that the 6-OHDA-induced neuronal apoptosis is mediated by the mitochondrial dysfunction. Markers of mitochondria function, such as mitochondrial membrane potential are often used to monitor the cell apoptosis. In the experiment, there was a significant reduction of MMP in 6-OHDA-treated SH-SY5Y cells. However, a partial restoration of MMP was observed in the cells treated with Wnt3a-CM at a proportion of 40 % (Fig. 4a and 4c). In consideration of ROS elevation believed to initiate a neurotoxic cascade induced by 6-OHDA [20], we further examined if Wnt3a-CM inhibit 6-OHDA-induced cell apoptosis by suppressing ROS production. As shown in Fig. 4b and d, 6-OHDA treatment alone for 24 h induced about 2-fold increase in ROS level compared to the control group, whereas pretreatment with 40 % Wnt3a-CM exhibited an inhibitive effect of ROS production from cells.

### Wnt3a-CM activated Wnt/β-catenin pathway

**Fig. 5:**
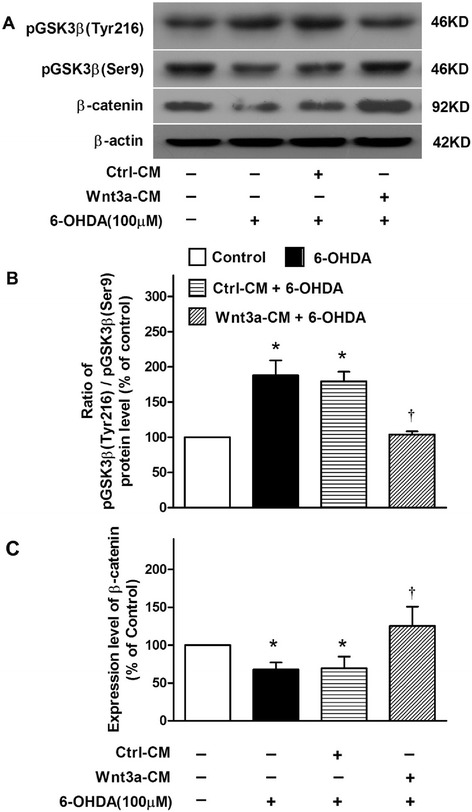
Change of related signal proteins in the cytoprotective effect of Wnt3a-CM in Sh-SY5Y treated with 6-OHDA. SH-SY5Y cells were treated with vehicle, 6-OHDA (100 μM), Ctrl-CM, or Wnt3a-CM (40 %) 20 min prior to 6-OHDA for 24 h, the protein levels of 2 phosphorylation forms of GSK3β and β-catenin (**a**) were detected by Western blot with β-actin as internal control. The band intensities were measured by Quantity One software and normalized to the expression of β-actin in SHSY5Y cells. The ratio of p-GSK3β (Tyr216) to p-GSK3β (Ser9) (**b**) and the relative levels of β-catenin (**c**) were expressed in histogram. Data were presented as mean ± SD of 3 experiments. *P < 0.05 compared to the control, †*P* < 0.05 compared to Ctrl-CM + 6-OHDA group

To verify the activation effect of Wnt3a-CM on Wnt/β-catenin pathway, western blot was used to detect the expression of p-GSK3β (Ser9), p-GSK3β (Tyr216) and β-catenin in SH-SY5Y cells. Because the activity of GSK3β is regulated negatively by the phosphorylation of serine 9 (Ser9) and positively by the phosphorylation of tyrosine 216 (Tyr216) [21], the ratio of p-GSK3β (Tyr216) to p-GSK3β (Ser9) can be used to monitor the activity of GSK3β. We found that treatment of 100 μM 6-OHDA for 24 h increase the ratio of p-GSK3β (Tyr216) to p-GSK3β (Ser9), and decrease β-catenin level. However, pretreatment with Wnt3a-CM reverse these changes induced by 6-OHDA treatment (Fig. 5a, b and c).

### Wnt3a-CM reversed 6-OHDA-induced Akt down-regulation

**Fig. 6:**
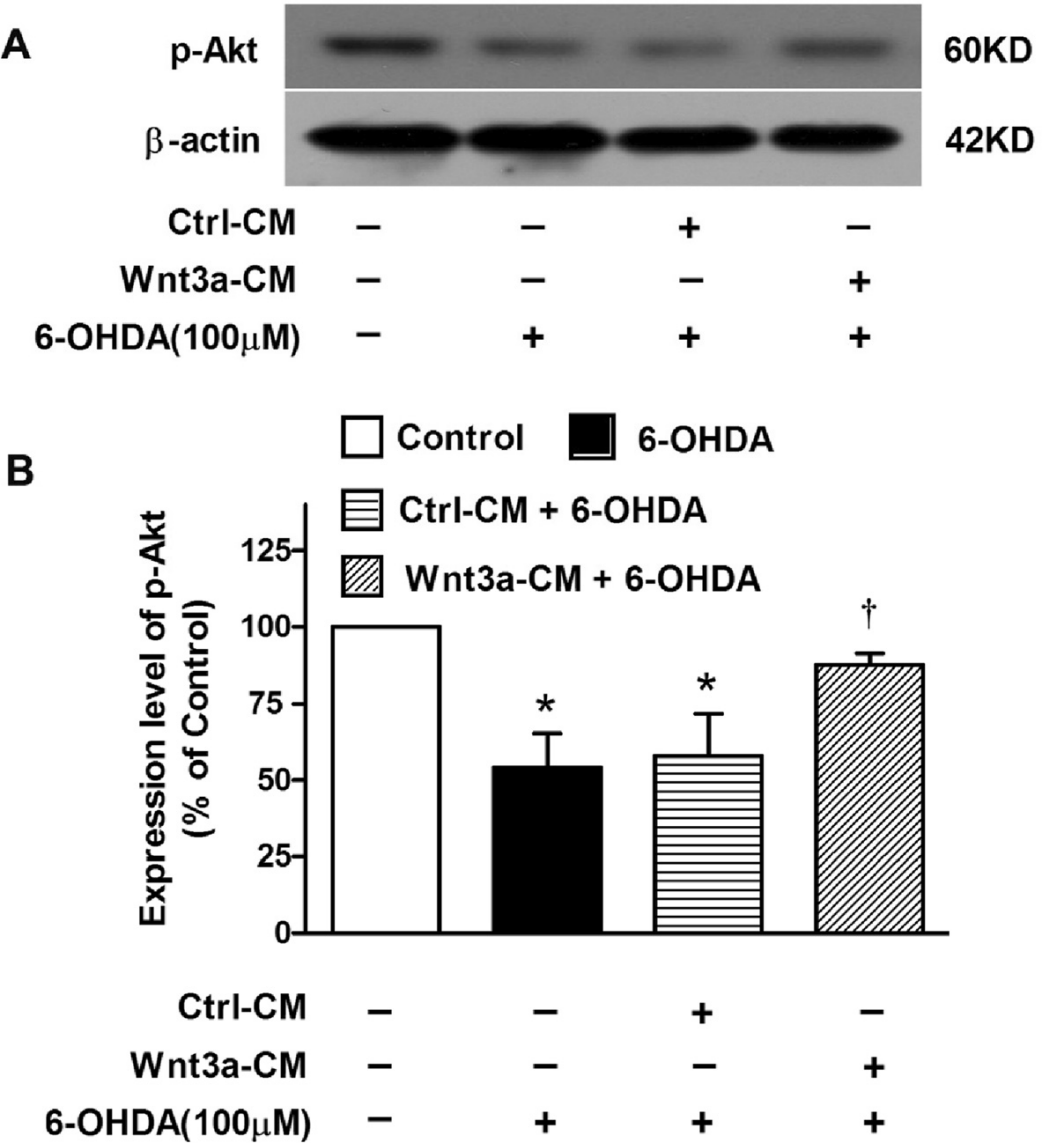
Change of p-Akt level in the cytoprotective effect of Wnt3a-CM in Sh-SY5Y treated with 6-OHDA. SH-SY5Y cells were treated with vehicle, 6-OHDA (100 μM), Ctrl-CM, or Wnt3a-CM (40 %) 20 min prior to 6-OHDA for 24 h, the protein levels of p-Akt (**a**) were detected by Western blot with β-actin as internal control. The relative band intensities of p-Akt (**b**) were measured by Quantity One software and normalized to the expression of β-actin in SHSY5Y cells. Data were presented as mean ± SD of 3 experiments. *P < 0.05 compared to the control, †*P* < 0.05 compared to Ctrl-CM + 6-OHDA group

Investigation has documented that Wnt signal rely upon PI3K/Akt activation to support the cell survival [22]. Here we detected the level of p-Akt (Ser473) protein, an active form of Akt, in SH-SY5Y cells treated with 6-OHDA or/and Wnt3a-CM. We found that the p-Akt protein level in 6-OHDA treated group were decreased to ~54 % compared with control group, while that in cells pretreatment of Wnt3a-CM was ~88 % (Fig. 6), which suggested that treatment of Wnt3a might reverse the down-regulation of PI3K/Akt pathway by 6-OHDA treatment.

## Discussion

This study investigated the protective effects of Wnt3a-conditioned medium on a cellular model of PD and showed that Wnt/β-catenin pathway was inhibited after treatment of 100 μM 6-OHDA for 24 h, evidenced by the decreased β-catenin level and increased GSK3β activity. Moreover, activating Wnt/β-catenin pathway by Wnt3a-CM was found to attenuate 6-OHDA-induced neurotoxicity in SH-SY5Y cells through restoration of mitochondria transmembrane potential and reducing ROS production, indicating the Wnt/β-catenin pathway related to the maintenance of mitochondrial function.

SH-SY5Y cell line chosen in this study was considered for its expression of tyrosine hydroxylase (TH) simulating to dopaminergic neurons [23]. As an endogenous oxidative metabolite of dopamine, 6-OHDA could inhibit the mitochondrial respiratory chain through inhibiting Complex I, uncoupling oxidative phosphorylation and collapsing mitochondrial membrane [24–26]. It is thought that 6-OHDA induces toxicity that mimics the neuropathological and biochemical characteristics of PD in SH-SY5Y cells [27].

Wnt3a is one of the Wnt ligands that activate the canonical Wnt pathway [28]. Because the purified Wnt3a is unstable, the Wnt3a conditioned medium is commonly used for the activation of the canonical Wnt pathway in in vitro experiments [29]. Recently, L’Episcopo et al. found the β-catenin protein acts as a pro-survival factor for mesencephalic TH^+^ neurons [14]. Meanwhile, Dickkopf-1 (DKK1), a negative regulator of the Wnt/β-catenin signaling pathway, was found to promote apoptosis of SH-SY5Y cells [30]. Dun Y and colleagues also found that induction of DKK1 contributes to the MPP^+^-induced neurotoxicity in PC12 cells via inhibition of the canonical Wnt pathway, and inhibition of DKK1 which could rescue the Wnt pathway might be neuroprotective in PD [31]. Our present study also confirmed that Wnt3a-CM increase the β-catenin protein level, which might contribute to the protective effect on cells. Moreover, our previous study uncovered that down-regulation of GSK3β, also a central component of the Wnt/β-catenin pathway, attenuate 6-OHDA-induced neuronal death and apoptosis [25]. The inhibition of GSK3β was reported to be linked with the attenuation of oxidative stress [32]. Data in this study confirmed that Wnt3a-CM inhibited the activity of GSK3β by increasing the phosphorylation at site Ser9 and decreasing the phosphorylation at site Tyr216.

Due to the mitochondrial dysfunction was considered a key factor in PD onset, we also measured MMP and ROS production from the SH-SY5Y cells in vitro. Mitochondria is the major site of ROS production and also prime target of oxidative molecular damage, the consequent formation of ROS further damages the mitochondrial membrane and such damages are implicated as key events in the pathogenic cascades leading to apoptosis [24, 33]. Shin SY and colleagues reported that stimulation of Wnt signaling by Wnt3a-CM inhibits H_2_O_2_-induced mitochondrial cytochrome C release and DNA fragmentation L1210 cells [34]. In this experiment, we found that Wnt3a-CM eliminated ROS production, stabilized mitochondrial transmembrane potential in SH-SY5Y cells.

PI3K and Akt are central to the regulation of cell growth and survival throughout the body [35, 36]. Akt, which is also known as protein kinase B (PKB), is a key molecule in growth factor signaling pathways mediating neuronal survival in both development and disease in multiple paradigms, including resistance against oxidative insults in the brain and protection of mitochondria function [37, 38]. Here our data showed that Wnt3a-CM reverse the down-regulation of p-Akt (Ser473) which is the active form of Akt caused by 6-OHDA treatment, clearly suggesting the mediation of the PI3K/Akt pathway in the protective effect of Wnt3a-CM on 6-OHDA-induced cell injury.

In conclusion, our data showed that activating of Wnt/β-catenin pathway by Wnt3a attenuated 6-OHDA-induced neurotoxicity, which involved in the mechanism about the inhibition of oxidative stress relate to the mitochondrial functional maintenance. These result may provide a new potential therapeutic target for Parkinson’s diseases.
